# Attention-Worthy Association: Prenatal Phthalate Exposure and Later Child Behavior

**DOI:** 10.1289/ehp.118-a172b

**Published:** 2010-04

**Authors:** Julia R. Barrett

**Affiliations:** **Julia R. Barrett**, MS, ELS, a Madison, WI–based science writer and editor, has written for *EHP* since 1996. She is a member of the National Association of Science Writers and the Board of Editors in the Life Sciences

Human exposure to phthalates is ubiquitous due to widespread commercial use. Although the compounds are reported to be rapidly metabolized, concentrations in the body appear to remain fairly stable due to ongoing exposure. The United States and Europe have banned some phthalates from consumer products primarily on the basis of reproductive toxicity data. However, not all phthalates are regulated; meanwhile, research indicates toxicity may extend to other endocrine targets such as the thyroid gland, which is critical for proper neurodevelopment. A new study now reports an association between prenatal exposure to certain phthalates and adverse effects on test scores used to evaluate children’s behavior and executive functioning **[*EHP*** **118:565–571; Engel et al.]**.

The prospective study was based on a multiethnic cohort of 404 women recruited during their first pregnancy for the Mount Sinai Children’s Environmental Health Study between 1998 and 2002. Each woman completed medical, sociodemographic, and lifestyle questionnaires and provided a urine sample between 25 and 40 weeks of pregnancy, which was used to measure phthalate metabolites. Metabolites were grouped according to their molecular weights.

When their children were approximately 4.5–5.5, 6–6.5, and 7–9 years old, 188 of the women completed the Behavior Rating Inventory of Executive Functioning (BRIEF) and the Behaviors Assessment System for Children-Parent Rating Scales (BASC-PRS), standardized forms used in clinical and research assessments of children’s executive functioning and behavior. Executive functions encompass planning to achieve goals, controlling attention and emotion, inhibiting inappropriate behaviors, and extrapolating from life experiences. Problematic behaviors assessed included hyperactivity, aggression, poor conduct, and issues with anxiety, attention, and adaptability.

High-molecular-weight phthalates—like those found in medical tubing and vinyl floor and wall coverings—were not associated with altered scores derived from the parent-report forms aside from a small association with reduced emotional control. However, low-molecular-weight phthalates—like those found in personal care products such as perfume, shampoo, cosmetics, and nail polish—were significantly associated with increased scores for aggression, attention and conduct problems, and depression.

The BASC-PRS includes a scale to help researchers weed out inaccurate assessments. The higher the resulting *F-*score, the more likely the assessment is to reflect an excessively negative evaluation of the child, a failure to follow instructions, random responding, or difficulty reading. When parent-report forms were restricted to those with *F-*scores of 0 or 1 (leaving 161 children in the analyses), most associations remained strong for boys but not girls. The sole exception was conduct problems, which remained significant for both girls and boys.

The behavioral problems assessed in this study are relevant to conditions such as oppositional defiant disorder, conduct disorder, and attention deficit/hyperactivity disorder. Diagnosing these conditions requires extensive testing beyond the scope of this study. Furthermore, this study cannot confirm that phthalate exposure caused these problems via altered thyroid function—or any other mechanism. However, thyroid-related phthalate toxicity makes a connection biologically plausible and underscores an urgent need to further investigate the effects of phthalates on neurodevelopment.

## Figures and Tables

**Figure:**
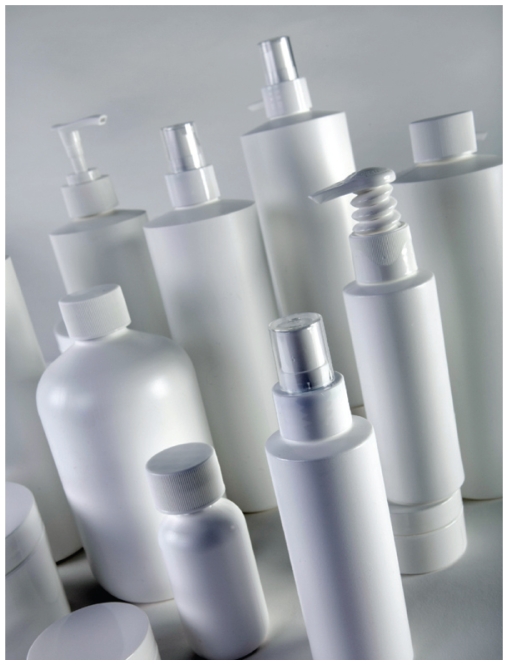
Low-molecular-weight phthalates are found in many personal care products.

